# Precision deletion of the entire coding sequence of the *mod-5* locus causes increase in pharyngeal pumping frequency

**DOI:** 10.17912/W2NP4D

**Published:** 2017-06-06

**Authors:** Trisha Brock, Stelian Pop, Chandler Bradford, Jenn Lawson, Lauren Resch, Christopher Hopkins

**Affiliations:** 1 Knudra Transgenics, 5201 S Green St., Salt Lake City, UT 84123, USA

**Figure 1.**
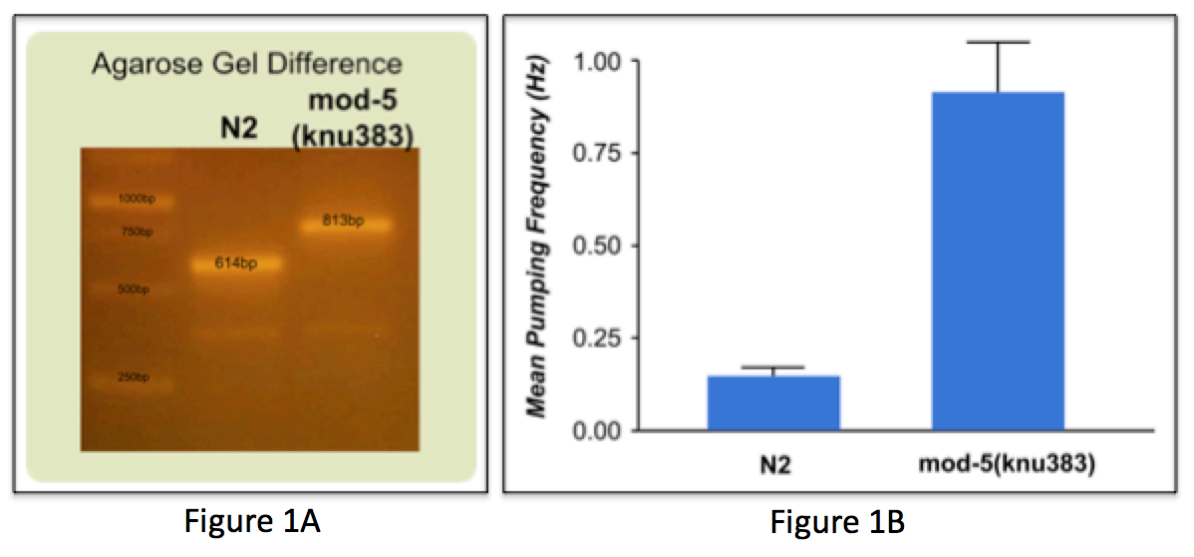


## Description

**​**The *C. elegans mod-5* gene encodes a serotonin transporter. The putative null allele contains a 1,688bp deletion leading to a premature stop codon in the resulting sequence (Ranganathan, 2001); however, unspliced the gene is 12,557bp in length from the start to the stop codon. We used CRISPR/Cas9 technology to create a 12,775bp deletion that eliminates all of the *mod-5* coding sequence from the genome (Figure 1A).

The *mod-5*(*knu383*) animals were measured for pharynx pumping frequency using the NemaMetrix ScreenChip (https://doi.org/10.17912/W2CC7Z; protocol described here http://www.knudra.com/p003). Compared with N2, *mod-5*(*knu383*) worms showed a statistically significant increase in pumping frequency (p<0.0001) (Figure 1B). For N2, 18 worms were tested in M9 with no stimulation, and 13 worms were tested for *mod-5*(*knu383*).

**Construction Details**The *mod-*5(*knu383*) allele repair oligonucleotide contained a 3-frame stop of TAAATAAATAAA surrounded by filler sequence of CCTCCCGTTCGCCTGGGACATC and GATGTCCCAGGCGAACGGGAGG. The homology arms were designed to give perfect homology for each of the sgRNA cut sites with 35 nucleotides of perfect homology on the 5’ side and 34 nucleotides on the 3’ side. These homology arms are 12,775bp apart from each other in the genomic sequence. The deletion was created using the CRISPR/Cas9 technology (Paix 2014, Kim 2014). The guide sequences were caaaagaaaagagcagccga and caaaagaaaagagcagccga provided in the injection mix as synthetic RNA. The deletion was detected by a three-primer PCR approach where an 813bp band would amplify in the deletion and a 614bp band would amplify in N2 wild-type, Figure 1A.

## Reagents

Strain: N2, COP1365 – *mod-5*(*knu383)* [12.8 kb entire coding-sequence deletion] NemaMetrix ScreenChip (Nemametrix).
